# Accuracy and precision of S_cv_O_2_ measured with the CeVOX-device: A prospective study in patients with a wide variation of S_cv_O_2_-values

**DOI:** 10.1371/journal.pone.0192073

**Published:** 2018-04-17

**Authors:** Alexander Herner, Bernhard Haller, Ulrich Mayr, Sebastian Rasch, Lea Offman, Roland Schmid, Wolfgang Huber

**Affiliations:** 1 Medizinische Klinik und Poliklinik II, Klinikum rechts der Isar, Technische Universität München, München, Germany; 2 Institut für Medizinische Statistik und Epidemiologie, Klinikum rechts der Isar, Technische Universität München, München, Germany; University of Bern, University Hospital Bern, SWITZERLAND

## Abstract

**Introduction:**

Central-venous oxygen saturation (S_cv_O_2_) is a key parameter of hemodynamic monitoring and has been suggested as therapeutic goal for resuscitation. Several devices offer continuous monitoring features. The CeVOX-device (Pulsion Medical Systems) uses a fibre-optic probe inserted through a conventional central-venous catheter (CVC) to obtain continuous S_cv_O_2_.

**Objectives:**

Since there is a lack of studies validating the CeVOX, we prospectively analyzed data from 24 patients with CeVOX-monitoring. To increase the yield of lower S_cv_O_2_-values, 12 patients were equipped with a *femoral* CVC.

**Methods:**

During the 8h study period S_cv_O_2__CeVOX was documented immediately before withdrawal of blood to measure S_cv_O_2_ by blood gas analysis (S_cv_O_2__BGA) 6min, 1h, 4h, 5h and 8h after the initial calibration. No further calibrations were performed.

**Results:**

In patients with *jugular* CVC (primary endpoint; 60 measurements), bias, lower and upper limits of agreement (LLOA; ULOA) and percentage error (PE) of the estimate of S_cv_O_2_ (S_cv_O_2__CeVOX_jug) were acceptable with 0.45%, -13.0%, 13.9% and 16.6%, respectively.

As supposed, S_cv_O_2_ was lower in the *femoral* compared to the *jugular* measurements (69.5±10.7 vs. 79.4±5.8%; p<0.001). While the bias (0.64%) was still acceptable, LLOA (-23.8%), ULOA (25.0%) and PE (34.5%) were substantially higher for *femoral* assessment of S_cv_O_2_ by the CeVOX (S_cv_O_2__CeVOX_fem).

Analysis of the entire data-pool with *jugular* as well as *femoral* CVCs allowed for a multivariate analysis which demonstrated that the position of the CVC per se was not independently associated with the bias S_cv_O_2__CeVOX—S_cv_O_2__BGA. The amount of the bias |S_cv_O_2__CeVOX–S_cv_O_2__BGA| was independently associated with the amount of the change of S_cv_O_2__CeVOX compared to the initial calibration to S_cv_O_2__BGA_baseline (|S_cv_O_2__CeVOX—S_cv_O_2__BGA_baseline|) as well as with low values of S_cv_O_2__BGA_baseline. Furthermore, increasing time to the initial calibration was associated to the amount of the bias with borderline significance.

A statistical model based on |S_cv_O_2__CeVOX—S_cv_O_2__BGA_baseline| and “time to last calibration” derived from an evaluation dataset (80 of 120 datasets, 16 of 24) provided a ROC-AUC of 0.903 to predict an amount of the bias |S_cv_O_2__CeVOX–S_cv_O_2__BGA| ≥5% in an independent validation group (40 datasets of 8 patients).

**Conclusion:**

These findings suggest that the CeVOX device is capable to detect stability or instability of S_cv_O_2__BGA. S_cv_O_2__CeVOX accurately estimates S_cv_O_2__BGA in case of stable values. However, intermittent measurement of S_cv_O_2__BGA and re-calibration should be performed in case of substantial changes in S_cv_O_2__CeVOX compared to baseline. Therefore, continuous measurement of S_cv_O_2_ with the CeVOX cannot replace S_cv_O_2__BGA in instable patients. On the other hand, CeVOX might be useful for the monitoring of stable patients as a pre-test tool for more differentiated monitoring in case of changes in S_cv_O_2__CeVOX.

## Introduction

In case of stable values for hemoglobin and the arterial oxygen saturation S_a_O_2_ the oxygen saturation of blood returning to the right heart necessarily depends on cardiac output (CO) and oxygen consumption VO_2_. The oxygen saturation of blood drawn from a central venous catheter (CVC) or via a pulmonary arterial catheter (PAC) has been termed central-venous oxygen saturation S_cv_O_2_ and mixed-venous oxygen saturation S_mv_O_2_, respectively. Both parameters reflect the relation of oxygen delivery and consumption. Normally, only about 25% of the delivered oxygen is withdrawn by the oxygen consuming tissues. Therefore, normal values for S_cv_O_2_ and S_mv_O_2_ are about 65–75%. In case of increased oxygen consumption and/or reduced delivery, S_cv_O_2_ and S_mv_O_2_ decrease. Consequently, decreasing values of S_cv_O_2_ and S_mv_O_2_ are used as warning signs indicating that mechanisms to compensate an impaired balance between oxygen consumption and delivery have been activated. While smaller decreases in S_cv_O_2_ and S_mv_O_2_ can be considered as physiological compensatory mechanism, more pronounced and prolonged decreases frequently precede anaerobic metabolism and hyperlactatemia.

Based on this pathophysiological rationale, S_cv_O_2_ and S_mv_O_2_ have been suggested as therapeutic goals for resuscitation and as key targets of hemodynamic monitoring to avoid tissue hypoxia despite normal macro-circulatory parameters such as MAP and CVP [1,2,3,4,5]. Furthermore, S_cv_O_2_ has been suggested as therapeutic goal for resuscitation and as a basic parameter of haemodynamic monitoring [3,6].

Several approaches have been established to facilitate these concepts:

Since the use of a PAC has certain risks and it is costly and limited in time, S_mv_O_2_ has largely been replaced by S_cv_O_2_ which can easily been determined via a CVC [1,2,3].

Since repeated measurements are cumbersome and costly in long term critically ill patients, several devices *continuously* deriving ScvO_2_ have been introduced. In addition to economic advantages, continuous monitoring offers the potential to increase the yield of pathological S_cv_O_2_ measurements. Therefore, continuous monitoring is in particular attractive in stable patients at risk of sudden circulatory instability to provide a sensitive pre-test tool for more differentiated monitoring.

Several devices offer continuous monitoring features, including the CeVOX (Pulsion Medical Systems SE, Feldkirchen, Germany). Usually, continuous measurement is based on infrared oximetry which detects transmitted light of different wavelengths reflected by red blood cells varying with different concentrations of oxyhemoglobin and hemoglobin [1].

While some devices have integrated the infrared probe into special catheters, the use of the CeVOX is even further facilitated, since the probe can be introduced into a catheter already in place. Despite its use for more than a decade, there are only few studies available that prove validity and clinical usefulness [7,8,9,10,11]. Some validation studies suggest that accuracy and precision might depend on the absolute value of S_cv_O_2_ with lower values resulting in imprecision compared to the gold-standard of blood gas analysis (BGA; Table 1).

**Table 1:** Experimental and clinical studies on the use of CeVOX.

Reference	Setting	No. of patients	No. of measurements	Mean and/or range ScvO2_BGA	Bias CeVOX–BGA	LLOA; ULOA	Comment
Huber D et al. [10]	In vitro	n.a.	2*40 (2 different CeVOX-catheters)	~60%; 9%-100%	+0.957%-0.175%	-7.69%; +9.61%-11.20%; +10.85%	In vitro using ECMO-device; underestimation of high S_cv_O_2_, overestimation of low S_cv_O_2_
Baulig W et al. [7]	In vitro	n.a.	n = 66	~55%;5.5–100%	+2.4%	-11.8%; +16.6%	In vitro using paediatric cardio-pulmonary bypass. Underestimation of high S_cv_O_2_, overestimation of low S_cv_O_2_
Baulig W et al. [8]	Cardiac surgery; ICU	n = 20	n = 84 surgery;	~70% (45%-89%)	-0.9%	-7.9%; +6.1%	Intra- and peri-operative measurements. Underestimation of high S_cv_O_2_, overestimation of low S_cv_O_2_. Underestimation in case of high cardiac index.
			n = 106 ICU	~75% (43%-90%)	-1.2%	-10.5%; +8.1%	
Müller M et al. [9]	Paediatric cardiac surgery	n = 3	n = 12	~60%; ~33–82%	-4.38%	-7.86%; -0.90%	4 measurements per patient
Molnar Z et al. [11]	Critically ill patients	n = 53	n = 526	72.3±9.9; ~30–95%	+0.3%	-12.5%; +13.2%	Multi-centric trial
This study: *jugular* CVC	Critically ill patients	n = 12	n = 60	79.4±5.7; 66–90%	+0.45%.	-13.0% +13.9%	
This study: *femoral* CVC	Critically ill patients	n = 12	n = 60	69.5±10.7; 33–86%	+0.64%	-23.8% +25.0%	Only study reporting on *femoral* CVC

Therefore, we performed a validation study in 24 patients equipped with the CeVOX device. To increase the yield of lower S_cv_O_2_-values, we included 12 patients equipped with a *femoral* CVC.

## Materials and methods

The study was approved by the institutional review board (Ethikkommission Technische Universität München; Fakultät für Medizin; No. 5384/12). Written informed consent was obtained by all patients or their legal representatives. All patients were treated in a 14-bed university hospital general ICU with predominantly medical patients. Informed consent was obtained by all patients or their legal representatives. Between July and October 2016 we included 24 patients with hemodynamic monitoring comprising transpulmonary thermodilution (TPTD; PiCCO; Pulsion Medical Systems SE, Feldkirchen, Germany), central venous catheter and measurement of S_cv_O_2_ irrespective of the study. For indicator injections for TPTD, blood withdrawal for blood gas analysis including S_cv_O_2_ and insertion of the CeVOX-probe (PV2022-37; Pulsion Medical Systems SE, Feldkirchen, Germany) a 5-lumen CVC (Multicath 5, Vygon; Aachen, Germany) with a maximum intravascular length of 20 cm and a diameter of 3.15 mm (9.5 Fr) was used. The position of the tip was controlled (and corrected) according to X-ray in case of jugular, but not in case of femoral venous catheter access. The vascular part of the femoral venous catheter was completely inserted under ultrasound guidance. The CeVOX was inserted into the medial lumen of the CVC ending at the tip of the catheter according to the manufacturer´s recommendations with the aim to protrude the distal end of the catheter by 2 cm. For insertion a sterile Y-adapter was used which enables insertion of the probe through one lumen and withdrawal of blood for BGA through the other lumen of adapter. This provides withdrawal of blood at the most distal lumen of the CVC in close proximity to the fiber probe. For blood gas analysis a Siemens RapidPoint 500 (Siemens Healthcare, Erlangen, Germany) analyzer was used. Baseline BGA was performed to calibrate the CeVOX subunit of a PiCCO-2 or Pulsioflex monitor (Pulsion Medical Systems SE, Feldkirchen, Germany). No further calibrations of the CeVOX were performed during the study period. To investigate a potential impact of cardiac index (CI) on the accuracy of the estimate of S_cv_O_2_ provided by the CeVOX (S_cv_O_2__CeVOX) a triplicate TPTD was performed immediately before the baseline BGA. The registration of the arterial TPTD curve was performed as previously described [12,13].

During the 8h study period S_cv_O_2__CeVOX was documented immediately before withdrawal of blood to measure S_cv_O_2_ by BGA (S_cv_O_2__BGA) 6min, 1h, 4h, 5h and 8h after the initial calibration.

### Statistical analyses

Raw data were examined for input data error. Continuous variables were expressed as mean±standard deviation. Categorical variables are expressed as percentages. Spearman´s coefficient of correlation was calculated to analyze the correlation of two parameters. To compare continuous variables we used Wilcoxon-test for paired samples.

Bland-Altman analysis was used to analyze the bias between S_cv_O_2__CeVOX and S_cv_O_2__BGA as well as to compute limits of agreement and percentage error. Bland-Altman analyses were corrected for repeated measurements allowing variability of true values within each subject [14].

With regard to clinical importance and comparability with previous studies Bland-Altman-analysis of the data derived from jugular BGA measurements was the primary endpoint of the study.

Prediction of the amount of the bias |S_cv_O_2__CeVOX–S_cv_O_2__BGA| was a major secondary endpoint. The amount of the bias is the absolute non-negative value of the bias without regard to its sign). To optimize the yield of our dataset, we used a three step approach: In a first step, we tried to assess the overall potential to derive a formula predicting inacceptable bias of S_cv_O_2__CeVOX. Therefore, we performed multivariate analysis regarding the amount of the bias |S_cv_O_2__CeVOX–S_cv_O_2__BGA| in the total dataset (n = 120) including variables with a p-value <0.2 in univariate analysis regarding this endpoint.

With regard to practical application only those variables were included in the regression analysis that would be available during continuous use of CeVOX after a single initial calibration.

In a second step, we randomly allocated the 24 patients in a 2:1 ratio to an evaluation group (n = 16 patients with 80 measurements) and to an independent validation group (n = 8 patients with 40 measurements). This was done to derive a prediction formula from two thirds of the datasets and to test its “robustness” in 40 “independent” measurements of the validation group that did not contribute to the derivation of the prediction formula (third step).

A similar approach has been described previously to derive and validate different models predicting inaccuracy of transpulmonary thermodilution with room temperature instead of ice-cold saline injectate [15].

Receiver operating characteristics (ROC)-analyses were performed to assess the discriminative ability of predictors regarding categorical endpoints.

Sample size was chosen according to the recommendation of Bland (https://www-user.york.ac.uk/~mb55/meas/sizemeth.htm). This publication suggests a number of n = 100 pairs in order to achieve an appropriate precision for the Bland-Altman analyses. Also accounting for potential drop-outs, incomplete datasets and pre-defined subgroup analyses we choose a number of n = 120.

All statistical analyses were performed using the IBM SPSS Statistics software version 23 (SPSS Inc., Chicago, IL, USA).

## Results

### Patients characteristics

Table 2 shows the patients baseline characteristics.

**Table 2:** Patients characteristics.

Based on datasets (n = 24)	
Sex (male:female; n (%))	13:11 (54%:46%)
Age (years±SD)	65±15
Underlying disease (n (%))	
- Sepsis	8 (33%)
- ARDS	12 (50%)
- Severe pancreatitis	2 (8%)
- Liver cirrhosis	2 (8%)
Height (cm ± SD)	173±9
Weight (kg ± SD)	81±20
APACHE-II score (n ± SD)	21±8
Measurements under vasopressors	16 (67%)
Measurements under mechanical ventilation	18 (75%)
Measurements under controlled ventilation (CV)	13 (54%)
Measurements under sinus rhythm (SR)	23 (96%)
Measurements under SR and CV	12 (50%)

All patients were critically with a mean APACHE-II score of 21. Half of the patients suffered from ARDS, another 33% of sepsis. Consequently, 75% were under mechanical ventilation and vasopressors were necessary during 67% of the measurements. The baseline cardiac index derived from TPTD with the PiCCO-device was 4±1.6L/min/m^2^.

### Comparison of S_cv_O_2__CeVOX_jug and S_cv_O_2__BGA_jug in patients with jugular CVC (primary endpoint)

In patients with *jugular* CVC (primary endpoint) S_cv_O_2__BGA_jug and S_cv_O_2__CeVOX_jug were significantly correlated (r = 0.567; p<0.001).

**Fig 1:**
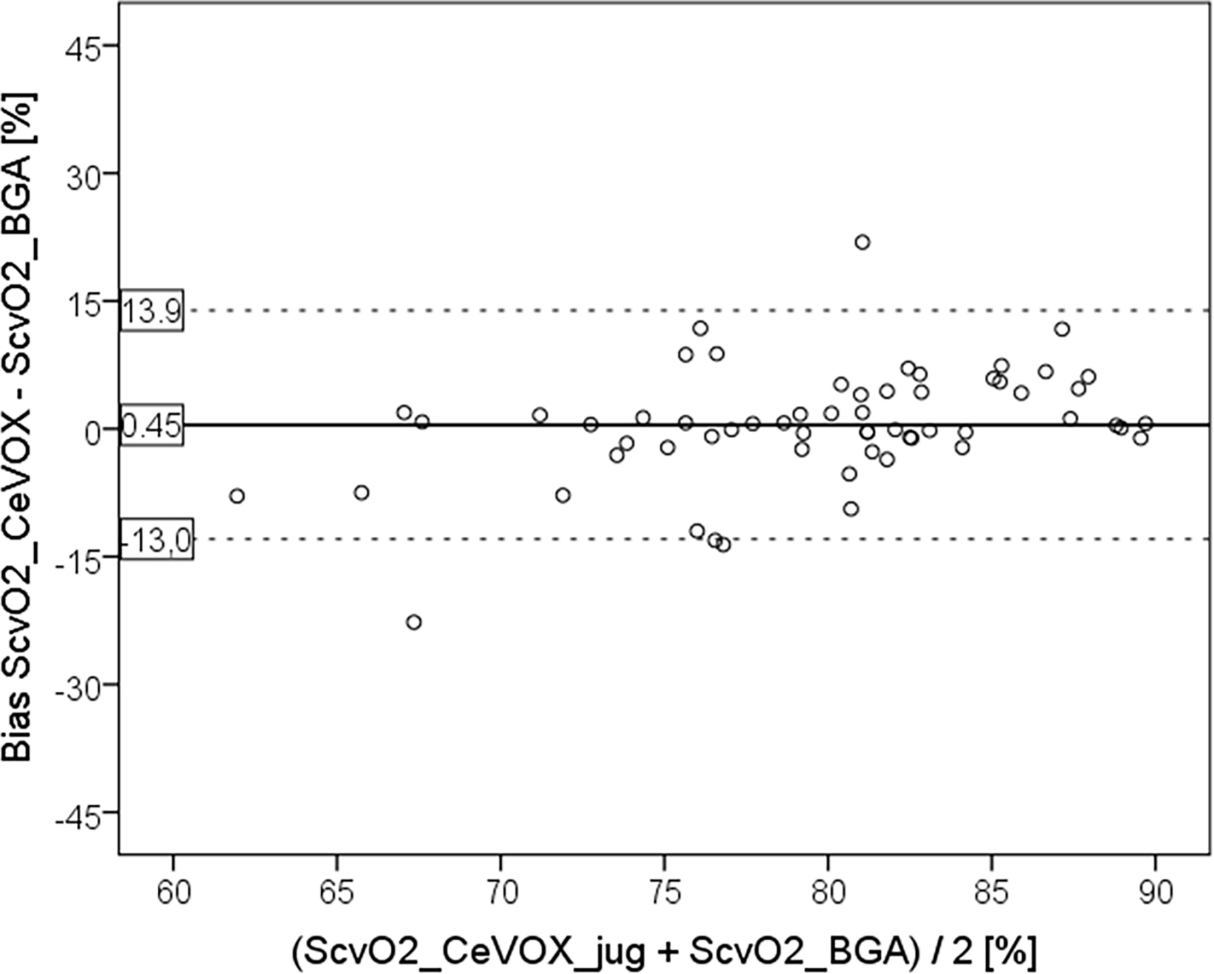
Bland Altman plot comparing S_cv_O_2__CeVOX_jug to S_cv_O_2__BGA derived from measurements with *jugular* CVC. S_cv_O_2__CeVOX_jug: Central venous oxygen saturation derived from the CeVOX-device. S_cv_O_2__BGA: Central venous oxygen saturation derived from blood gas analysis. CVC: Central venous catheter.

S_cv_O_2__CeVOX_jug and S_cv_O_2__BGA_jug were not significantly different (79.9±8.2 vs. 79.4±5.7%; p = 0.337) with a mean bias of 0.45±6.8%. Lower and upper limits of agreement (LLOA; ULOA) and percentage error (PE) were acceptable with -13.0%, 13.9% and 16.6% respectively (Fig 1).

### Comparison of S_cv_O_2__CeVOX_fem and S_cv_O_2__BGA_fem in patients with femoral CVC

As supposed, S_cv_O_2__BGA was lower in *femoral* compared to *jugular* measurements (69.5±10.7 vs. 79.4±5.8%; p<0.001).

Similar to jugular measurements S_cv_O_2__BGA_fem and S_cv_O_2__CeVOX_fem significantly correlated (r = 0.488; p<0.001) for measurements with *femoral* vein CVC access. Mean values of S_cv_O_2__CeVOX_fem and S_cv_O_2__BGA_fem were comparable (70.1±13.1 vs. 69.5±10.7%; p = 0.496) with a mean bias of 0.64%.

**Fig 2:**
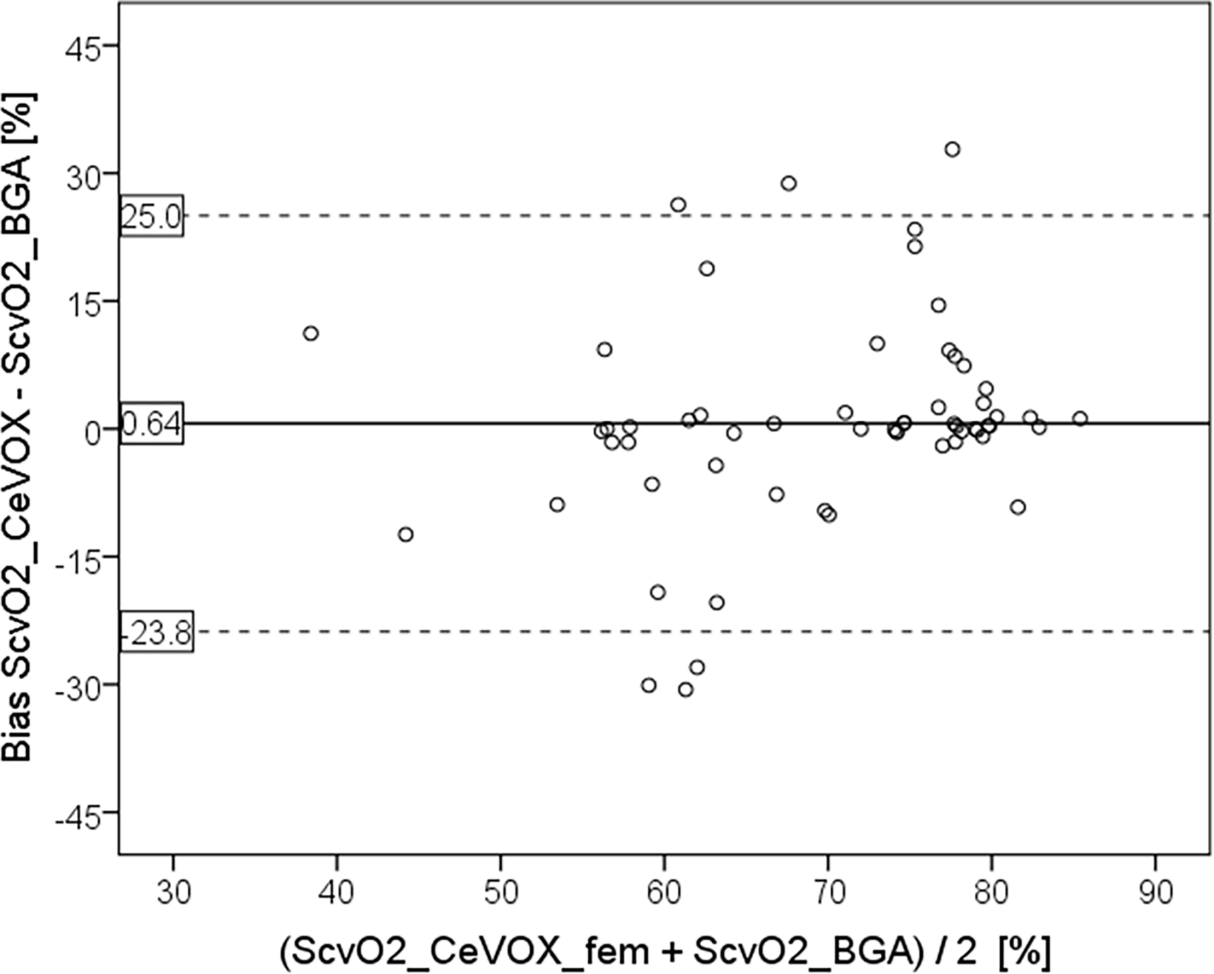
Bland Altman plot comparing S_cv_O_2__CeVOX_fem to S_cv_O_2__BGA derived from measurements with *femoral* CVC. S_cv_O_2__CeVOX_fem: Central venous oxygen saturation derived from the CeVOX-device. S_cv_O_2__BGA: Central venous oxygen saturation derived from blood gas analysis. CVC: Central venous catheter.

While the bias was still acceptable, LLOA (-23.8%), ULOA (25.0%) and PE (34.5%) were substantially higher for femoral assessment of S_cv_O_2_ by the CeVOX (S_cv_O_2__CeVOX_fem; Fig 2) compared to S_cv_O_2__CeVOX_jug.

### Analyses with jugular and femoral CVC

To further analyze the potential impact of catheter position (jugular or femoral), the amount of S_cv_O_2__BGA, time to calibration of the CeVOX-device and other variables on accuracy and precision of S_cv_O_2__CeVOX we analyzed the total dataset including jugular as well as femoral catheter positions.

**Fig 3:**
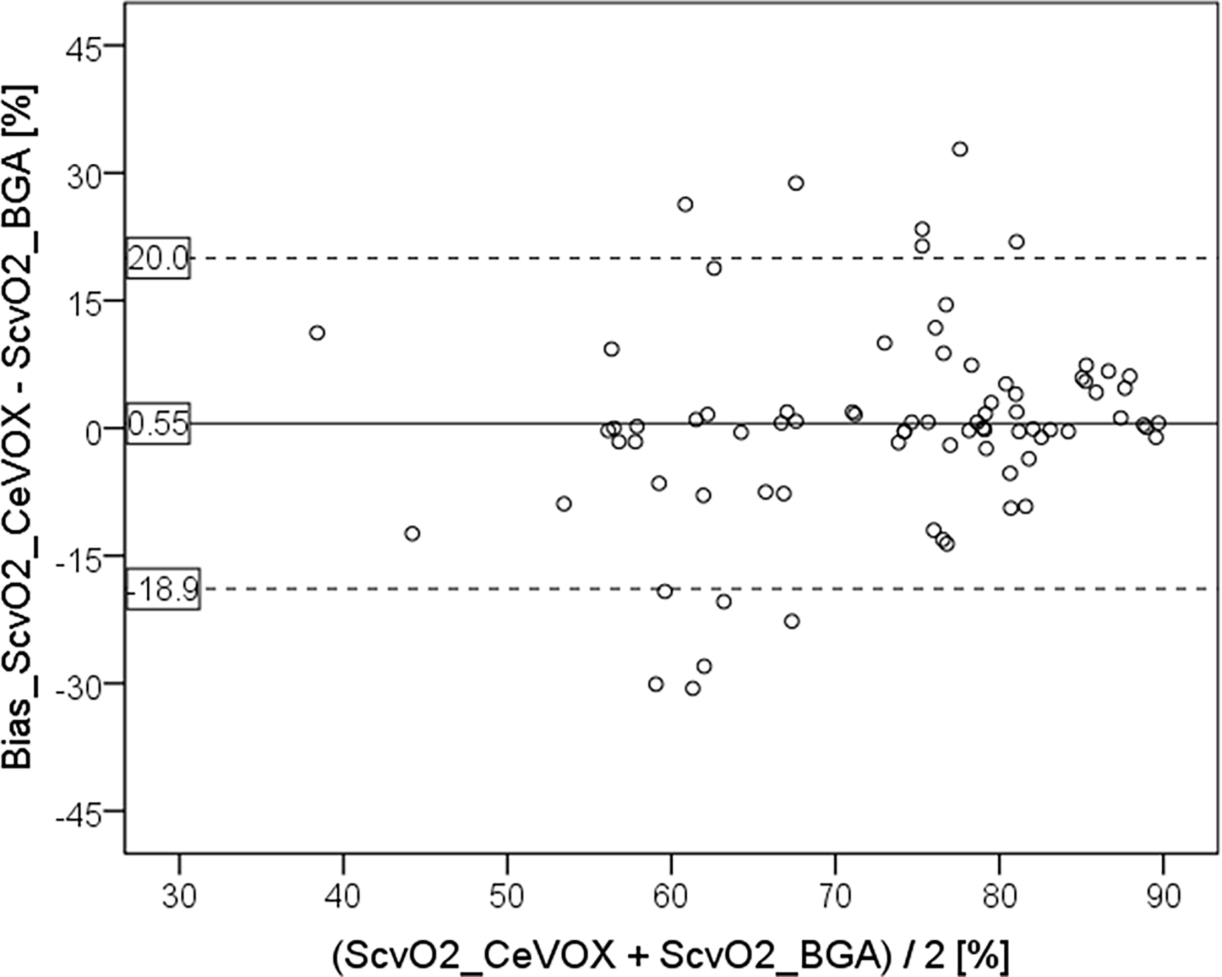
Bland Altman plot comparing S_cv_O_2__CeVOX to S_cv_O_2__BGA derived from all measurements (jugular or femoral CVC). S_cv_O_2__CeVOX: Central venous oxygen saturation derived from the CeVOX-device. S_cv_O_2__BGA: Central venous oxygen saturation derived from blood gas analysis. CVC: Central venous catheter.

S_cv_O_2__BGA and S_cv_O_2__CeVOX significantly correlated (r = 0.607; p<0.001). Mean values of S_cv_O_2__CeVOX and S_cv_O_2__BGA were comparable (75.0±11.9 vs. 74.5±9.9%; p = 0.262) with a mean bias of 0.55%, LLOA of -18.9%, ULOA of 20.0% and a PE of 25.9% (Fig 3).

### Predictors of imprecision of S_cv_O_2__CeVOX in all patients with jugular or femoral CVC

Table 3 shows the univariate correlations (Spearman) of several variables to the bias S_cv_O_2__CeVOX–S_cv_O_2_ and to the amount of the bias |S_cv_O_2__CeVOX–S_cv_O_2__BGA|. The bias and its amount were neither correlated with *femoral* position of the CVC nor with the base CI derived from TPTD.

**Table 3:** Univariate association of different variables to S_cv_O_2__CeVOX–S_cv_O_2_ and the amount |S_cv_O_2__CeVOX–S_cv_O_2_|.

Variable	Association to bias S_cv_O_2__CeVOX–S_cv_O_2__BGA		Association to the amount |S_cv_O_2__CeVOX–S_cv_O_2__BGA|	
	r-value	p-value	r-value	p-value
CVC_femoral	r = -0.008	p = 0.931	r = 0.041	p = 0.659
Time after calibration	r = 0.018	p = 0.846	**r = 0.561**	**p<0.001**
S_cv_O_2__BGA	r = -0.160	p = 0.082	**r = -0.243**	**p = 0.008**
S_cv_O_2__CeVOX	**r = 0.556**	**p<0.001**	r = -0.086	p = 0.355
BGA_baseline	r = -0.056	p = 0.545	**r = -0.226**	**p = 0.014**
CI_td_baseline	r = -0.140	p = 0.147	r = -0.045	p = 0.640
PEEP_base	**r = -0.280**	**p = 0.006**	r = 0.029	p = 0.780
PIP_base	r = 0.056	p = 0.593	r = 0.046	p = 0.662
F_i_O_2__base	r = -0.120	p = 0.250	r = 0.149	p = 0.151
pH	r = -0.096	p = 0.300	r = 0.102	p = 0.272
pCO_2_	**r = 0.262**	**p = 0.004**	r = -0.157	p = 0.088
pO_2_	r = -0.141	p = 0.129	**r = -0.249**	**p = 0.007**
HCO_3_^-^	r = 0.113	p = 0.220	r = -0.013	p = 0.884
Tricuspid regurgitation [grade]	r = 0.067	p = 0.499	r = 0.02	r = 0.799
Δ_S_cv_O_2__BGA—S_cv_O_2__BGA_baseline	**r = -0.323**	**p<0.001**	**r = -0.257**	**p = 0.005**
Δ_S_cv_O_2__CeVOX—S_cv_O_2__BGA_baseline	**r = 0.790**	**p<0.001**	r = 0.028	p = 0.763
|(Δ_S_cv_O_2__BGA—S_cv_O_2__BGA_baseline)|	r = 0.076	p = 0.410	**r = 0.532**	**p<0.001**
| (Δ_S_cv_O_2__CeVOX—S_cv_O_2__BGA_baseline)|	r = 0.038	p = 0.685	**r = 0.773**	**p<0.001**

In univariate analysis bias S_cv_O_2__CeVOX–S_cv_O_2__BGA was significantly associated with changes in S_cv_O_2_ compared to baseline S_cv_O_2__BGA.

The bias S_cv_O_2__CeVOX–S_cv_O_2__BGA was associated with high values of S_cv_O_2__CeVOX (r = 0.556; p<0.001), high values of Δ_ScvO_2__CeVOX—S_cv_O_2__BGA_baseline (r = 0.790; p<0.001) and lower values of Δ_S_cv_O_2__BGA—S_cv_O_2__BGA_baseline (r = -0.323; p<0.001). Furthermore, the bias S_cv_O_2__CeVOX–S_cv_O_2__BGA was associated with lower PEEP (r = -0.280; p = 0.006) and higher central-venous pCO_2_ (r = 0.262; p = 0.004) with a low degree of correlation.

The amount of the bias |(S_cv_O_2__CeVOX–S_cv_O_2__BGA)| was univariately associated with increasing time after calibration (r = 0.561; p<0.001), lower values of S_cv_O_2__BGA (r = -0.242; p = 0.008), lower values of S_cv_O_2__BGA_baseline (r = -0.226; p = 0.014) and of Δ_S_cv_O_2__BGA—S_cv_O_2__BGA_baseline (r = -0.257; p = 0.005). A particular strong association of the amount of the bias |(S_cv_O_2__CeVOX–S_cv_O_2__BGA)| was found for the absolute changes in S_cv_O_2__BGA and S_cv_O_2__Cevox compared to S_cv_O_2__BGA_baseline (r = 0.773; p<0.001 for |(Δ_S_cv_O_2__CeVOX—S_cv_O_2__BGA_baseline)| and r = 0.532; p<0.001 for |(Δ_S_cv_O_2__BGA—S_cv_O_2__BGA_baseline)|. This suggests that the amount of the bias increases with increasing changes of S_cv_O_2_ (measured by BGA or by CeVOX) compared to baseline.

Furthermore, the bias |(S_cv_O_2__CeVOX–S_cv_O_2__BGA)| was weakly associated with a lower central-venous pO_2_ (r = -0.249; p = 0.007).

Close association of one or more parameters to (the amount of) the bias S_cv_O_2__CeVOX–S_cv_O_2__BGA could be of interest for practical use, e.g. to provide an internal control for the device suggesting recalibration by measurement of S_cv_O_2__BGA as a kind of recalibration-alarm.

Therefore, we performed multivariate analysis regarding the amount of the bias |S_cv_O_2__CeVOX–S_cv_O_2__BGA|. With regard to practical application only those variables were included in the regression analysis that would be available during continuous use of CeVOX after a single initial calibration.

For this major secondary endpoint we used a three-step-approach:

In a first step we performed a multiple regression analysis in the total dataset (n = 120) regarding the amount of the bias |S_cv_O_2__CeVOX–S_cv_O_2__BGA|.

**Fig 4:**
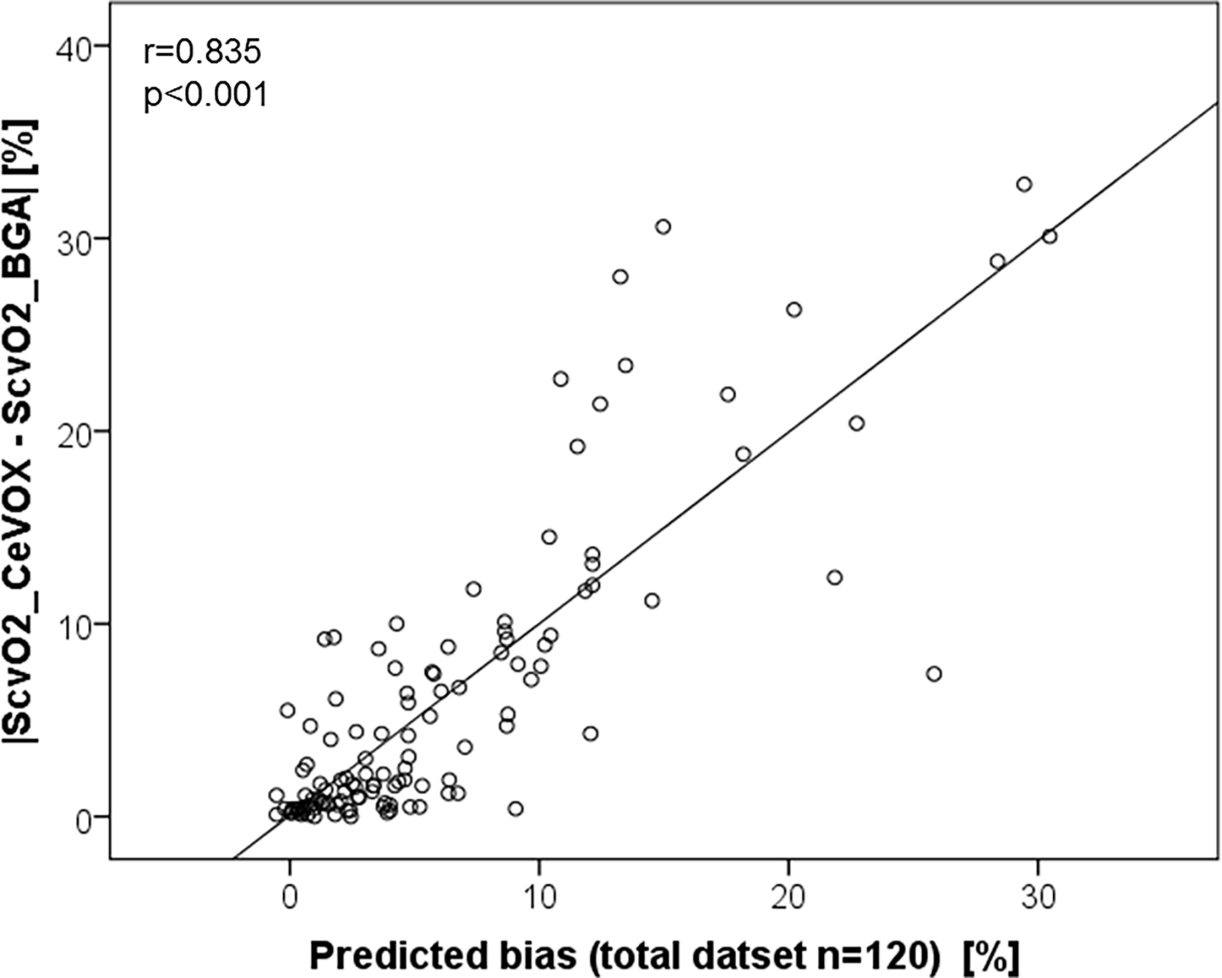
Correlation of the amount of the bias |S_cv_O_2__CeVOX–ScvO2_BGA| with a prediction formula derived from the total datset (n = 120). S_cv_O_2__CeVOX: Central venous oxygen saturation derived from the CeVOX-device. S_cv_O_2__BGA: Central venous oxygen saturation derived from blood gas analysis.

The final model (R = 0.835; R^2^ = 0.697; Fig 4) included |S_cv_O_2__CeVOX–S_cv_O_2__BGA_baseline| (p<0.001; t-value14.959), S_cv_O_2__BGA_baseline (p = 0.007; t = -2.770) and–with borderline significance–S_cv_O_2__CeVOX (p = 0.083; t-value = 1.751, whereas time after initial calibration and CVC-site were not independently associated to the amount of the bias |S_cv_O_2__CeVOX–S_cv_O_2__BGA|.

In a second step, we randomly divided the 120 datasets in 80 evaluation datasets and 40 independent validation datasets.

The final model derived from the 80 evaluation datasets included |S_cv_O_2__CeVOX—S_cv_O_2__BGA_baseline| (p<0.001; t-value 11.610) as outstanding predictor of |S_cv_O_2__CeVOX–S_cv_O_2__BGA|. Furthermore, “time to last calibration” was independently associated with |S_cv_O_2__CeVOX–S_cv_O_2__BGA| with borderline significance (p = 0.065; t-value 1.875) in this model. The predictive capabilities of this model were high (R = 0.830; R^2^ = 0.689).

**Fig 5:**
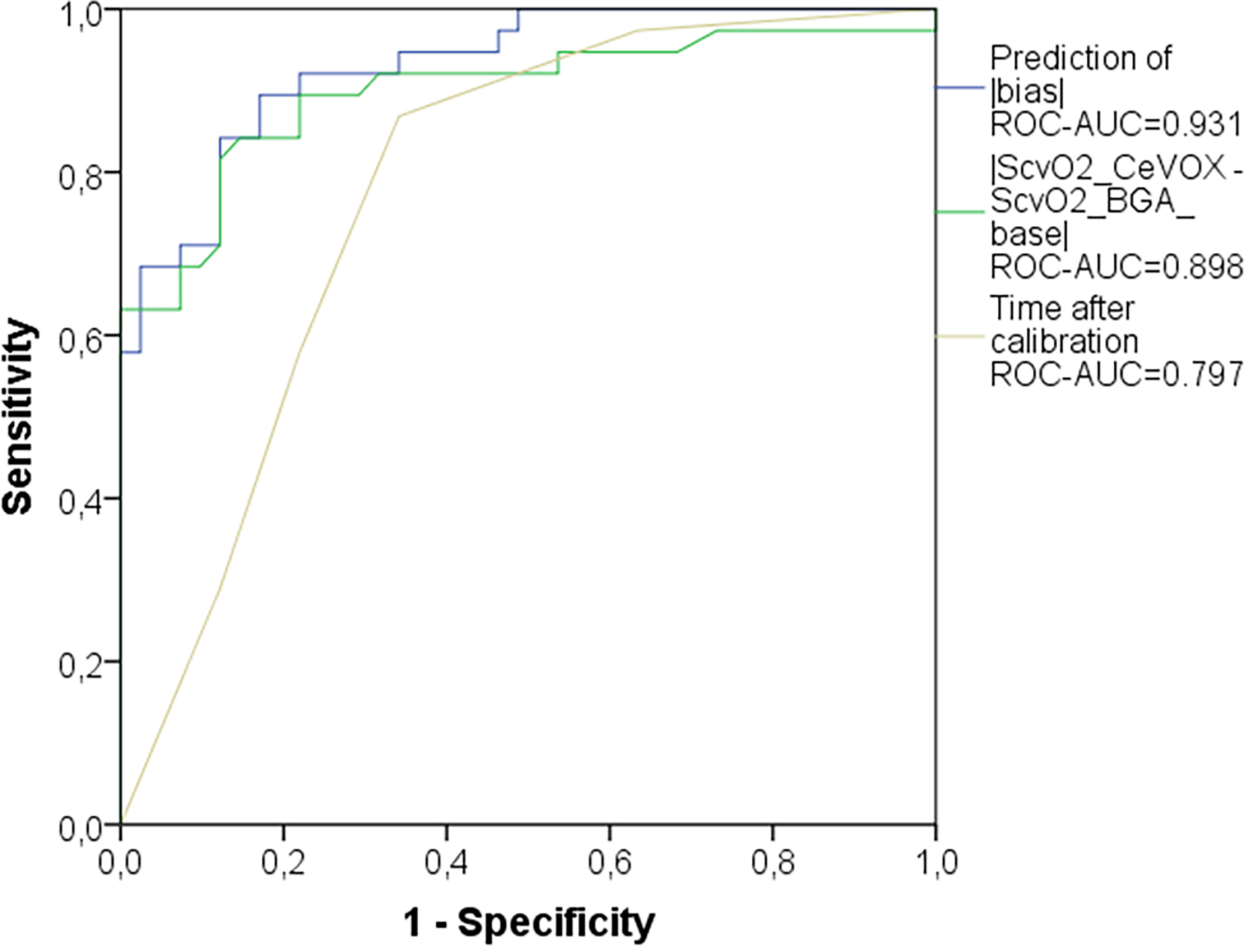
ROC curve comparing different predictors of |S_cv_O_2__CeVOX–S_cv_O_2__BGA| ≥5% in the *evaluation* group (n = 80). “Prediction of |bias|”: model predicting |S_cv_O_2__CeVOX–S_cv_O_2__BGA| which was derived from multiple regression analysis within the evaluation group. ROC: receiver operating characteristic. AUC: area under the curve. S_cv_O_2__CeVOX: Central venous oxygen saturation derived from the CeVOX-device. S_cv_O_2__BGA: Central venous oxygen saturation derived from blood gas analysis.

A prediction model derived from these two parameters provided a ROC-AUC of 0.931 (p<0.001) in the evaluation group to predict an amount of the bias |S_cv_O_2__CeVOX–S_cv_O_2__BGA| ≥5%. The ROC-AUC for this model was slightly higher than that for |S_cv_O_2__CeVOX—S_cv_O_2__BGA_baseline| (AUC = 0.898; p<0.001) and substantially higher than for time to last calibration (AUC = 0.779; p<0.001; Fig 5).

**Fig 6:**
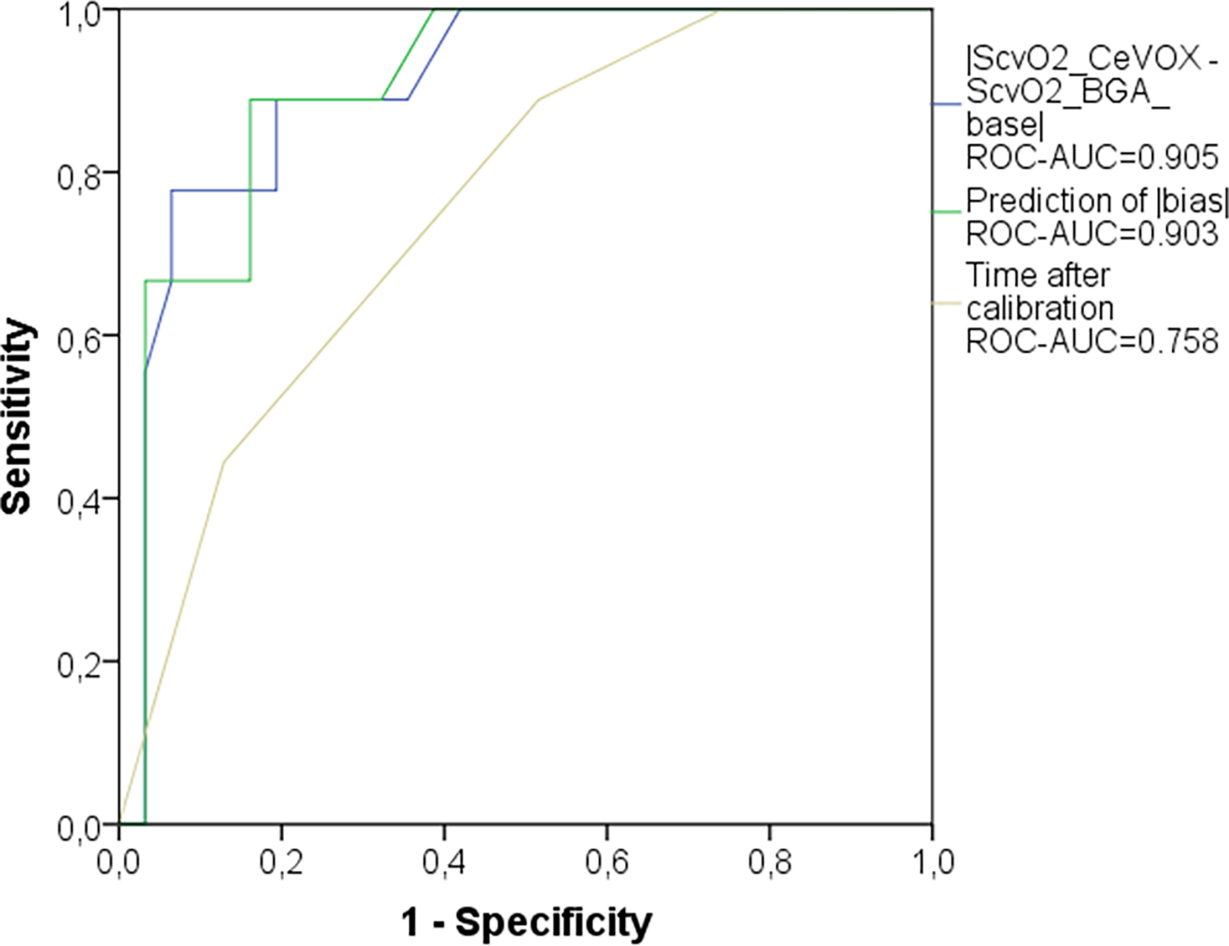
ROC curve comparing different predictors of |S_cv_O_2__CeVOX–S_cv_O_2__BGA| ≥5% in the independent *validation* group (n = 40). Prediction of |bias|: model predicting |S_cv_O_2__CeVOX–S_cv_O_2__BGA| which was derived from multiple regression analysis within the *evaluation* group. ROC: receiver operating characteristic. AUC: area under the curve. S_cv_O_2__CeVOX: Central venous oxygen saturation derived from the CeVOX-device. S_cv_O_2__BGA: Central venous oxygen saturation derived from blood gas analysis.

In a third step, the prediction model derived from the *evaluation* group provided a ROC-AUC of 0.903 (p<0.001) in the *validation* group to predict an amount of the bias |S_cv_O_2__CeVOX–S_cv_O_2__BGA| ≥5%. This was in the same range as for |ScvO2_CeVOX—ScvO2_BGA_baseline| (AUC = 0.905; p<0.001), but substantially higher than for “time after last calibration” (AUC = 0.758; p = 0.020; Fig 6).

## Discussion

S_cv_O_2_ has been used to guide resuscitation with [3] and without success [16,17,18]. Most of the patients in the intervention group in these studies were equipped with a CVC with *continuous* S_cv_O_2_ monitoring capability. Despite the use of continuous measurement of S_cv_O_2_ and S_mv_O_2_ for several decades, there are only few studies available that prove their accuracy, precision and clinical usefulness.

This also applies to the CeVOX-device. Our observational study compared S_cv_O_2__CeVOX to S_cv_O_2__BGA during an 8-hours period without recalibration, but repeated withdrawal of blood to determine S_cv_O_2__BGA. We deliberately included patients with a *femoral* CVC to increase the yield in lower values of S_cv_O_2_, since in critically ill patients with (analgo)-sedation S_cv_O_2_ in vena cava inferior usually is lower than in vena cava superior.

Using this approach the following main results were found:

1. For higher values of S_cv_O_2__BGA derived from jugular CVCs (primary endpoint) the S_cv_O_2__CeVOX_jug provided acceptable bias, percentage error and limits of agreement.
2. By contrast, for lower values of S_cv_O_2_ predominantly withdrawn from femoral CVCs, S_cv_O_2__CeVOX_fem provided an acceptable bias, but inappropriately high values for the percentage error and the limits of agreement as well as a poor correlation to S_cv_O_2__BGA (r = 0.488).
3. Analysis of the entire data-pool with *jugular* as well as *femoral* CVCs allowed for the multivariate analysis which demonstrated that the position of the CVC per se was not independently associated with the bias S_cv_O_2__CeVOX—S_cv_O_2__BGA. The amount of the bias was independently associated with the amount of the change of S_cv_O_2__CeVOX compared to the initial calibration to S_cv_O_2__BGA_baseline as well as to low values of S_cv_O_2__BGA_baseline. Furthermore, increasing time to the initial calibration was associated to the amount of the bias with borderline significance.
4. A statistical model based on |S_cv_O_2__CeVOX—S_cv_O_2__BGA_baseline| and “time to last calibration” derived from an evaluation dataset (80 of 120 datasets, 16 of 24) provided a ROC-AUC of 0.903 to predict an amount of the bias |S_cv_O_2__Cevox–S_cv_O_2__BGA| ≥5% in an independent validation group (40 datasets of 8 patients).

As for most of the few previously published studies the bias for S_cv_O_2__CeVOX was acceptable and clearly within a range between -1% and +1%, irrespective of jugular (+0.45%) or femoral (+0.63%) position of the CVC [7,8,9,10,11].

In case of *jugular* CVC, lower and upper limits of agreement (-13,0%; +13.9%) for S_cv_O_2__CeVOX_jug were in the range of the two previous in vitro and of three in vivo studies with nearly identical values as for the study by Molnar and colleagues including patients from the same ICU as this study (-12.5; +13.2%; see Table 1).

However, for lower S_cv_O_2__CeVOX_femderived from a *femoral* CVC LLOA (-23.8%) and ULOA (+25.0%) were not in the acceptable range. Consequently, percentage error values were acceptable for jugular measurements (16.6%), “borderline” for the totality of measurements (25.9%) and out of the acceptable range for femoral measurements (34.5%). At first glance, even the percentage error for femoral measurements seems in the same range as given by Molnar et al. (35.5%). However, a closer look at this study demonstrates that the percentage error was calculated by dividing the difference between ULOA and LLOA by the mean of S_cv_O_2__CeVOX and S_cv_O_2__BGA. This is an unusual method to calculate the percentage error which results in values twice as high as suggested by Critchley and colleagues [19]. Consequently, the percentage error according the Critchley-method would have been 17.8% in the Molnar-study which is the range of our findings for jugular measurements.

Our findings of a lower precision of S_cv_O_2__CeVOX_fem in case of lower S_cv_O_2__BGA derived from femoral measurements raise the key question, if imprecision is related to femoral measurement, to lower values of S_cv_O_2__BGA or to any other variables. With a wide range of S_cv_O_2__BGA between 33% and 90%, jugular as well as femoral measurements, different standardized intervals between measurements and concomitant TPTD measurement of CI our data allowed for *univariate* as well as *multivariate* analyses of different potential confounders of S_cv_O_2__CeVOX.

Several previous studies (see Table 1) suggested a *systematic* imprecision resulting in an underestimation of high and an *over*estimation of high values of S_cv_O_2__BGA [7,8,10]. By contrast, in our study there was a better agreement for higher values of S_cv_O_2_ in general without a hint for a systematic *under*estimation.

One of five previous studies demonstrated an underestimation of S_cv_O_2__BGA by the CeVOX-device in case of high cardiac index [8]. These findings were neither confirmed by our univariate nor by multivariate analyses. However, the study by Baulig et al. was performed in patients undergoing cardiac surgery with a markedly lower mean CI of 2.6 (range 1.0–4.5 L/min/m^2^) compared to our study (mean CI 4.3±1.6; range 2.1–7.4 L/min/m^2^).

On the contrary, our results with increasing imprecision of CeVOX with lower values of S_cv_O_2__BGA are in line with the statement of Molnar et al. that “the scatter increases as S_cv_O_2_ goes below 65%” [11].

Although our study suggests that imprecision of the CeVOX is associated rather to low values of S_cv_O_2_ than to *femoral* measurement per se, a potential impact of femoral measurement has to be discussed: Kissoon and co-workers demonstrated in an animal experiment simulating various hemodynamic conditions that oximetry-based estimates of S_cv_O_2_ derived with a different catheter were more imprecise for measurements in the inferior vena cava than in the superior vena cava. They concluded that “the oximetry catheter is capable of identifying changes in S_cv_O_2_ under physiological conditions usually encountered in clinical medicine but was less accurate at the extremes of physiology and when placed in the inferior vena cava catheter especially during hypovolaemia and hypoxia” [20].

Finally, “drifts over time” have been reported [10], suggesting decreasing precision over time. These findings are supported by our study showing uni- and multivariate association of the amount of the bias to the time to last calibration. This clearly suggests the need for regular re-calibration of continuous oximetry catheters estimating S_cv_O_2_.

According to our analyses of the total data pool as well as of separate evaluation and validation groups changes in S_cv_O_2__CeVOX compared to the baseline calibration were–by far–the strongest predictors of imprecision of the S_cv_O_2__CeVOX.

### Practical implications

Derivation of a formula—based on changes in S_cv_O_2__CeVOX compared to calibration and on time to last calibration—predicting inappropriate precision of S_cv_O_2__CeVOX might have practical implications. Since the usefulness of this formula was confirmed in an independent validation group of patients, this formula could be implemented as a kind of automated quality control suggesting re-calibration. A similar “calibration-index” has been suggested for re-calibration of continuous pulse contour analysis derived cardiac index by transpulmonary thermodilution [21]. A modification of the suggested formula has been included in the latest software of the PiCCO-2-device as a kind of decision support to optimize re-calibration and to improve the yield of relevant findings by thermodilution.

### Strengths and limitations

Heterogeneity of the patients, also investigating measurements derived from femoral CVCs increasing the yield of low values of S_cv_O_2_, availability of TPTD to measure CI can be considered as strengths of the study. These characteristics also allowed for the multivariate analyses. Furthermore, the number of patients and measurements was sufficient to validate findings within an *evaluation* group in an independent *validation* group.

Finally, the findings of the study might have practical implications with regard to the above-mentioned “calibration-index” as a kind of automated quality control which might improve patient care and safe personal as well as material resources.

Nevertheless, this single-center study is based on a limited number of patients and measurements over a short period.

### Conclusions

Continuous estimation of S_cv_O_2_ by the CeVOX is accurate and precise, if the values are close to those at baseline. However, re-calibration should be performed in case of substantial changes compared to baseline. Furthermore, low values of S_cv_O_2_ and longer time to calibration were associated with imprecision of the CeVOX-device. The integration of a calibration.index indicating the need for recalibration might help to improve the precision of the CeVOX device.

Finally, continuous measurement of S_cv_O_2_ with the CeVOX cannot replace S_cv_O_2__BGA in instable patients. On the other hand, CeVOX might be useful for monitoring of stable patients as a pre-test tool for more differentiated monitoring in case of changes in S_cv_O_2__CeVOX. Improved continuous assessment of S_cv_O_2_ including automated quality control might be useful also during anaesthesia in high risk surgery and during the stabilization phase in certain patients in an accident and emergency department.
